# Synergistic LaCoO_3_@Co_3_O_4_ bifunctional catalyst for efficient oxygen evolution and reduction: achieving low polarization

**DOI:** 10.1039/d5ra09353h

**Published:** 2026-04-22

**Authors:** Akihito Shio, Hayato Takada, Yuna Fujiwara, Taketo Imamura, Toshiki Iwato, Kouki Yamamoto, Gasidit Panomsuwan, Takahiro Ishizaki

**Affiliations:** a Materials Science and Engineering, Graduate School of Engineering and Science, Shibaura Institute of Technology Tokyo 135-8548 Japan; b Department of Materials Engineering, Faculty of Engineering, Kasetsart University Bangkok 10900 Thailand; c College of Engineering, Shibaura Institute of Technology Tokyo 135-8548 Japan ishizaki@shibaura-it.ac.jp

## Abstract

Lithium–oxygen batteries (LOBs) suffer from low cycle stability and limited capacity primarily due to the sluggish kinetics of the oxygen evolution reaction (OER) and the oxygen reduction reaction (ORR). To address this and develop high-performance air cathodes, we systematically synthesized a series of metal oxide catalysts (Co_3_O_4_ (CO), LaCoO_3_ (LCO), and LaCoO_3__Co_3_O_4_ (LCO_CO)) using a coprecipitation method and evaluated their bifunctional catalytic activities for OER and ORR in an alkaline electrolyte. Among the synthesized materials, the LCO_CO composite catalyst demonstrated superior bifunctional activity, achieving the lowest potential gap (Δ*E*) of 1.14 V between the OER and ORR. Intriguingly, this exceptional performance was achieved despite LCO_CO exhibiting the lowest electrochemical surface area (ECSA) value. These findings strongly indicate that the catalytic performance was governed not by the macroscopic quantity of the surface area, but by qualitative factors such as the structure and electronic state of the atomic-level active sites (*i.e.*, intrinsic activity). Structural analyses by XRD and XPS suggested that the homogeneous compounding, coupled with the incorporation of Co^2+^ ions during synthesis, induced defects (possibly La site vacancies), leading to a significant increase in lattice oxygen vacancies and an optimization of the Co^3+^/Co^2+^ ratio. Electronic structure investigations further revealed that these synergistic structural and electronic modifications narrowed the energy gap between the Co 3d and O 2p band centers, thereby reducing the charge transfer energy. This modulation not only optimizes the adsorption of oxygenated intermediates but also facilitates the participation of lattice oxygen *via* the Lattice Oxygen Mechanism (LOM). We concluded that these interfacial synergistic structural and electronic modifications critically facilitated electron transfer during the OER/ORR processes, contributing to the observed exceptional performance. This study provides crucial guidance for designing high-performance composite oxide catalysts for LOBs, emphasizing that qualitative control of active site structure and electronic coupling is key over purely quantitative surface area maximization.

## Abstract 2

A synergistic LaCoO_3_@Co_3_O_4_ composite catalyst achieved a low potential gap of 1.14 V. Interfacial lattice defects and optimized electronic states facilitated the lattice oxygen mechanism, enhancing bifunctional OER/ORR activity.

## Graphical abstract

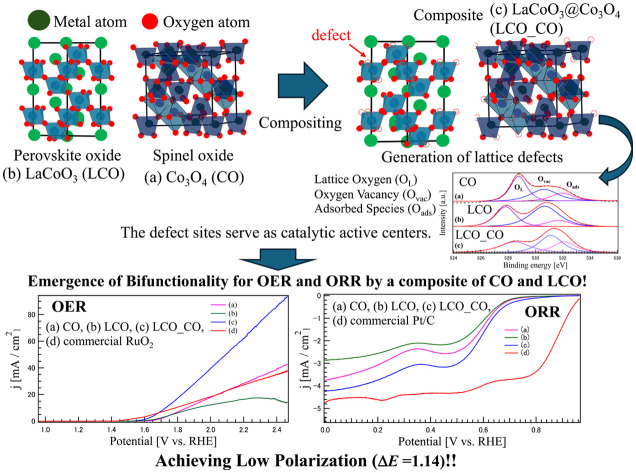

## 1 Introduction

The rapid evolution of the mobility industry, particularly drones and electric vehicles (EVs), necessitates the urgent development of high-energy-density batteries capable of surpassing the theoretical limitations of current lithium-ion batteries (LIBs).^1^ While LIBs typically offer an energy density of approximately 200–300 Wh kg^−1^, achieving the future target of 500 Wh kg^−1^ requires the realization of next-generation energy storage systems. Among these, the Lithium–Oxygen Battery (LOB) stands out due to its exceptionally high theoretical energy density, leveraging a lithium metal anode and atmospheric oxygen as the active cathode material.^1–3^ The fundamental operation of LOBs revolves around the reversible electrochemistry occurring at the air cathode: oxygen reduction reaction (ORR), which leads to discharge and the formation of lithium peroxide (Li_2_O_2_), and the subsequent oxygen evolution reaction (OER) during charging, which decomposes the Li_2_O_2_.^1,3^ The overall performance of an LOB is critically dependent on the catalytic activity of the material coated on the cathode current collector.^1,4–6^ Despite their promise, the practical implementation of LOBs is hindered by several major challenges, including short cycle life, failure to meet the 500 Wh kg^−1^ energy density goal, and difficulties in achieving high-rate charge/discharge due to limitations in oxygen diffusion and cathode reaction kinetics.^2,7,8^ Previous research has primarily utilized carbon materials as the main cathode scaffold due to their high electrical conductivity and Li_2_O_2_ accommodation capacity.^1,2,7^ To enhance the sluggish ORR/OER kinetics, significant attention has been focused on incorporating catalysts, particularly metal oxides such as perovskite and spinel oxides.^8–17^ These oxide catalysts, like the spinel Co_3_O_4_, are highly effective in LOBs.^13–16^ Their superior catalytic function is attributed to the ability of doping (aliovalent substitution) to modify the electronic state by altering the bond strength and configuration (bond angle, lattice strain, and oxygen vacancies) between the B-site atoms and oxygen, which in turn optimizes the adsorption of reactants onto the surface.^17–19^ Lanthanum (La) was chosen as the A-site cation for the perovskite phase due to its ability to stabilize the BO_6_ octahedral network and its role in tailoring the oxidation state of cobalt, which is critical for efficient oxygen adsorption/desorption processes.^20^ In recent years, composite catalysts integrating perovskite and spinel structures have garnered increasing interest for improving the efficiency of lithium–oxygen batteries, as such transition metal oxide heterostructures are recognized as promising bifunctional air-cathode materials.^21,22^ Based on these findings, it is hypothesized that the complexation (or hybridization) of different oxide structures can lead to a substantial synergistic enhancement in ORR/OER activity that is unattainable with single-component oxides. This represents the core motivation of our study. However, a major challenge in synthesizing such composite materials is achieving compositional homogeneity between the two distinct oxide phases. To address this, the coprecipitation method is an effective synthesis route, as it enables the simultaneous and uniform crystal growth of both components from a precursor solution.^23^

While the individual catalytic activities of perovskite and spinel oxides are well-established, the synergistic effect of their intimate combination, especially when prepared *via* a highly homogeneous method, remains largely unexplored for LOB applications. Therefore, this study aims to synthesize a novel composite metal oxide, LaCoO_3__Co_3_O_4_, by the coprecipitation method, combining the highly anticipated perovskite LaCoO_3_ with the known high-performing spinel Co_3_O_4_. The physicochemical properties of the resulting composite were systematically analyzed, and its ORR and OER characteristics against the individual single-component oxides were comparatively evaluated, thereby providing critical insights into the design of high-performance catalysts for rechargeable LOBs.

## 2 Experimental methods

### 2.1 Synthesis of composite and single metal oxides^23^

The LaCoO_3__Co_3_O_4_ (LCO_CO) composite was synthesized using a coprecipitation method. First, 4 mmol of CoCl_2_ (97.0%, Fujifilm Wako Pure Chemical Corporation) and 1 mmol of La(NO_3_)_3_·6H_2_O (99.0%, Fujifilm Wako Pure Chemical Corporation) were dissolved in a mixed solvent consisting of 30 ml of deionized water and 5 ml of ethanol. Subsequently, 6 ml of a 2 M NaOH solution was added to the mixture, and the resulting solution was stirred for 1 h to obtain the precursor precipitate. The solid product was collected by vacuum filtration and then introduced into a muffle furnace. The temperature was raised to 750 °C at a rate of 5 °C min^−1^ and held at this temperature for 12 h to yield the final LCO_CO composite. The synthesis conditions, including the calcination temperature and duration, were chosen based on preliminary experiments to ensure the formation of highly crystalline perovskite and spinel phases without significant particle sintering. Furthermore, the molar ratio of LCO to CO was optimized by synthesizing a series of composites with different La : Co ratios (1 : 1, 1 : 3, and 1 : 5). Preliminary electrochemical evaluations revealed that the 1 : 3 molar ratio (designated as LCO_CO) exhibited the most superior bifunctional catalytic activity (see SI, S1, Fig. S1, S2, and Table S1). Consequently, this optimized composition was selected for further in-depth structural characterization and mechanistic studies. For comparative analysis, single-phase LCO and CO were synthesized using a similar precipitation and calcination procedure. The precursor for LCO was prepared by mixing 1 mmol of CoCl_2_ and 1 mmol of La(NO_3_)_3_·6H_2_O in a mixed solvent of 30 ml deionized water and 5 ml ethanol, followed by the addition of 6 ml of 2 M NaOH and 1 h of stirring. The resulting precipitate was collected *via* vacuum filtration and calcined in a muffle furnace at 750 °C for 12 h with a heating rate of 5 °C min^−1^. The precursor for CO was obtained by dissolving 2 mmol of CoCl_2_ in the mixed solvent (30 ml of deionized water and 5 ml of ethanol), adding 6 ml of 2 M NaOH, and stirring for 1 h. The collected precursor was subsequently calcined in a muffle furnace at 750 °C for 12 h with a heating rate of 5 °C min^−1^.

### 2.2 Characterization of synthesized oxides

The crystal structure, morphology, chemical composition, and specific surface area of the synthesized materials were characterized. The crystal phases of the samples were identified using a CuKα X-ray Diffractometer (Smart Lab, Rigaku Corporation). Measurements were performed in the *θ*–2*θ* mode, covering a 2*θ* range of 10 to 90° with a scanning speed of 10.0° min^−1^. The shape and morphology of the materials were observed using a field emission scanning electron microscope (FE-SEM, JSM-7610F, JEOL Co. Ltd). Elemental composition analysis was performed using energy dispersive X-ray spectroscopy (EDS) attached to the SEM system. The chemical binding states and qualitative composition were analyzed using X-ray photoelectron spectroscopy (JPS-9010MC, JEOL, Co., Ltd). XPS measurements were conducted using AlKα radiation with an applied voltage of 10 kV and a current of 25 mA. The C 1s peak at 284.5 eV was used for charge-up correction of the obtained spectra. The specific surface area and pore size distribution were determined using a TriStar II 3020 analyzer (Micromeritics) *via* the nitrogen adsorption/desorption method. Before measurement, an appropriate amount of each sample was degassed under vacuum at 100 °C for 24 h. The measurement was carried out with the container filled with liquid nitrogen. All linear sweep voltammetry (LSV) curves for OER were recorded at a scan rate of 0.01 V s^−1^. The measured potentials *vs.* Ag/AgCl were converted to the RHE scale using the Nernst equation: *E*_RHE_ = *E*_Ag/AgCl_ + 0.197 + 0.059 × pH.

### 2.3 Electrochemical performance

The catalytic activities for the ORR and OER were evaluated using a dual electrochemical analyzer (ALS704ES, BAS, Co.) with a rotating ring–disk electrode (RRDE) system (RRDE-3A, BAS, Co.). The catalyst ink (slurry) was prepared by dispersing 5 mg of the synthesized sample in a mixture of 480 µL of deionized water, 480 µL of ethanol, and 40 µL of Nafion® solution (5 wt%, Sigma-Aldrich) using ultrasonic agitation. 7.5 µL of the prepared slurry was drop-cast onto a Pt(ring)–GC(disk) electrode (glassy carbon disk diameter: 4 mm, *A*_disk_ = 0.126 cm^2^; inner/outer-ring diameter: 5.0/7.0 mm, *A*_ring_ = 0.188 cm^2^) and dried overnight under ambient pressure. A standard three-electrode cell configuration was used, employing the catalyst-coated Pt(ring)–GC(disk) as the working electrode, a Pt wire as the counter electrode, and an Ag/AgCl electrode saturated with KCl solution as the reference electrode. The electrolyte was a 0.1 M KOH aqueous solution. For ORR and OER measurements, the electrolyte was first purged with nitrogen gas for 20 min to remove dissolved oxygen and then saturated with oxygen by purging O_2_ gas. Measurements were conducted at a rotation rate of 1500 rpm. For the ORR measurements, linear sweep voltammetry (LSV) was conducted within a potential window of 0 to −1.0 V (*vs.* Ag/AgCl) at a scan rate of 10 mV s^−1^. Conversely, the OER activity was evaluated by scanning the potential from 0 to 1.5 V (*vs.* Ag/AgCl) at a scan rate of 10 mV s^−1^. The electrocatalytic activities for ORR and OER were primarily evaluated using LSV in O_2_-saturated 0.1 M KOH. Although the background capacitive currents were found to be negligible compared to the high faradaic currents observed during the oxygen electrode reactions, the catalytic performance was rigorously quantified based on the well-defined onset and half-wave potentials. The OER activity was quantified by the overpotential (*η*_10_) required to reach a current density of 10 mA cm^−2^ (*j* = 10 mA cm^−2^).

The electrochemically active surface area (ECSA) was estimated by measuring the double-layer capacitance in the potential range of 0.2 to 0.3 V *vs.* Ag/AgCl in 1 M KOH aqueous solution saturated with oxygen, using the cyclic voltammetry (CV) technique. The potential sweep rates used were 0.05, 0.1, 0.2, 0.3, 0.4, and 0.5 V s^−1^. The details of the determination of ECSA and specific activity are described in SI as S2. All potentials measured against the Ag/AgCl (saturated KCl) reference electrode were converted to the reversible hydrogen electrode (RHE) scale using the Nernst equation: *E*_RHE_ = *E*_Ag/AgCl_ + 0.197 + 0.059 × pH, where *E*^0^_Ag/AgCl_ is 0.197 V at 25 °C. In this study, for 1 M KOH electrolyte (pH ≈ 14), the conversion is *E*_RHE_ = *E*_Ag/AgCl_ + 1.023 V. Overpotentials (*η*) for OER were calculated using the relation *η* = *E*_RHE_ − 1.23 V.

## 3 Results and discussion

### 3.1 Crystalline structure analysis

**Fig. 1:**
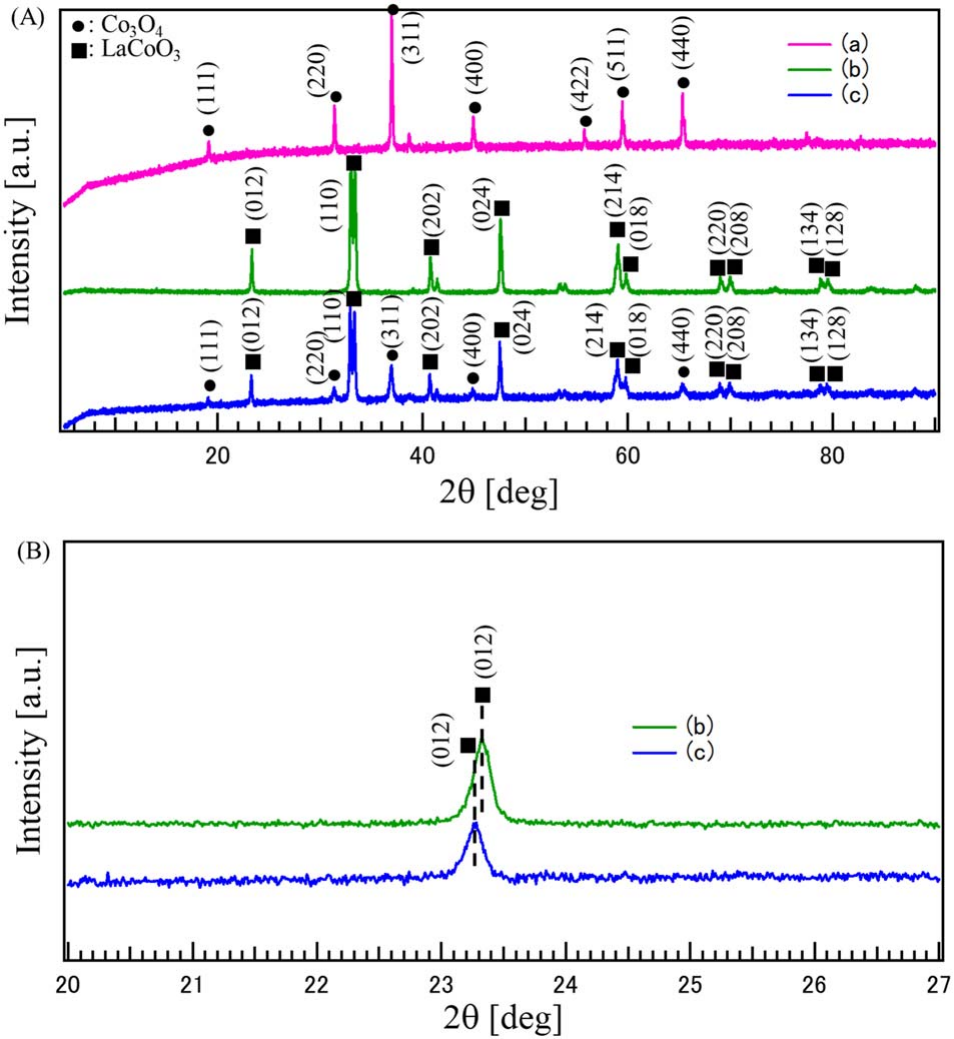
(A) XRD patterns of the catalysts synthesized by the co-precipitation method: (a) CO, (b) LCO, and (c) LCO_CO composite. (B) Magnified view of the (012) reflection peak of (b) LCO and (c) LCO_CO.

Fig. 1(A) shows the X-ray diffraction (XRD) patterns of the synthesized samples: (a) CO, (b) LCO, and (c) the composite (LCO_CO). The XRD pattern of (b) LCO exhibits characteristic diffraction peaks belonging to the perovskite oxide LaCoO_3_ (ICDD-PDF: 00-025-1060). These peaks are observed at 2*θ* = 23.27, 32.91, 40.66, 47.52, 59.01, 68.98 and 69.94°, which are indexed to the (012), (110), (202), (024), (214), (220), and (208) reflections, respectively. The XRD pattern of (a) CO confirms the formation of the spinel oxide Co_3_O_4_ (ICDD-PDF: 00-042-1467). Characteristic peaks are observed at 2*θ* = 19.00, 31.27, 36.85, 44.81, 55.66, 59.36, and 65.24°, corresponding to the (111), (220), (311), (422), (400), (511), and (440) planes of the spinel structure. The XRD pattern of the (c) LCO_CO composite clearly shows the coexistence of all major characteristic peaks from both the LCO and CO single phases, confirming the successful formation of the LaCoO_3__Co_3_O_4_ composite without any detectable secondary phases beyond the intended components. To determine the precise phase composition of the composite, Rietveld refinement (WPPF) was performed on the XRD pattern of LCO_CO (Fig. S3). As summarized in Table S2, the weight ratio of the LCO phase to the CO phase was calculated to be 67.3 : 32.7, with good fitting parameters (*R*_wp_ = 4.04%, *R*_p_ = 2.86%, *S* = 1.24). Although the synthesis was performed with a cobalt-rich nominal stoichiometry, these quantitative results indicate that the LCO phase is the predominant crystalline component in the final composite. A closer inspection of the XRD pattern for (c) LCO_CO in the 2*θ* range of 20 to 27° reveals a peak shift compared to the single LCO phase (b). Specifically, the main LCO (012) peak is slightly shifted to a lower angle (a clearer demonstration is shown in the magnified region of Fig. 1(B)). This shift indicates an expansion of the *d*-spacing for the (012) plane, which strongly suggests the presence of lattice strain within the synthesized composite material.^15^ This phenomenon is hypothesized to arise from the misalignment of atomic arrangements at the grain boundaries between the perovskite (LCO) and spinel (CO) phases, which is induced by the crystallographic mismatch between the two structures. Consequently, this lattice strain may lead to the formation of a distorted structure and, potentially, the inducement of oxygen vacancy formation, which is often associated with enhanced catalytic function in metal oxides.^9,18^

### 3.2 Morphological analysis

**Fig. 2:**
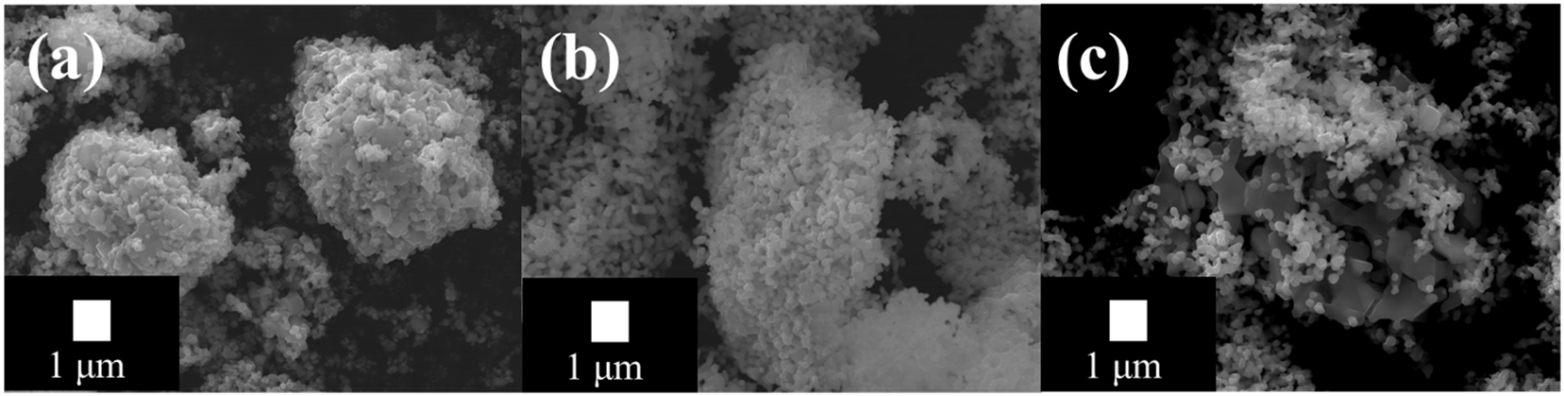
SEM images of the catalysts synthesized by the coprecipitation method: (a) CO, (b) LCO, and (c) LCO_CO composite.

Fig. 2 presents the high-resolution FE-SEM images of (a) CO, (b) LCO, and (c) LCO_CO at the same magnification with a 1 µm scale bar for direct comparison. All samples exhibit a particulate morphology composed of primary particles (∼100 nm to sub-micrometer levels). Crucially, the primary particle morphology of the LCO_CO composite remains largely unchanged compared to the precursors, confirming that no significant morphological degradation or uncontrolled grain growth occurred during the coprecipitation synthesis. While the composite appears to form larger secondary agglomerates, the consistent BET results (Table 1) confirm that the overall porosity and accessible surface are preserved, indicating that the primary particles are not fused but are instead assembled into porous clusters.

**Table 1:** BET specific surface area measurement results for each sample

Sample	Specific surface area [m^2^ g^−1^]
(a) CO	20.8
(b) LCO	22.8
(c) LCO_CO	24.6

Direct microscopic evidence of the intimate interfacial contact between the LCO and CO phases within these agglomerates is provided by the STEM-EDS mapping (Fig. S4). The mapping reveals a highly uniform and overlapping distribution of La, Co, and O at the nanoscale, confirming that the two phases are homogeneously integrated without significant phase separation. This high-density contact at the hetero-interfaces is essential for generating the interfacial boundary effects—such as lattice strain and a high concentration of oxygen vacancies (41.3% for LCO_CO, as summarized in Table 2)—which are critical for maximizing synergistic catalytic effects. As illustrated in Fig. 11, these structural defects modulate the electronic structure by narrowing the energy gap between the Co 3d and O 2p band centers. This electronic modulation facilitates the Lattice Oxygen Mechanism (LOM) and enhances the bifunctional performance by decreasing the charge transfer energy. Consequently, the intrinsic activity gain from these defect-rich interfaces significantly outweighs the macro-scale clustering observed in the SEM analysis.

**Table 2:** Relative peak area ratios of O 1s components (O_L_, O_vac_, and O_ads_) derived from XPS deconvolution for CO, LCO, and LCO_CO composite samples

Sample	Lattice oxygen (O_L_) [%]	Oxygen vacancy (O_vac_) [%]	Adsorbed species (O_ads_) [%]
CO	45.9	35.3	18.8
LCO	34.9	52.0	13.1
LCO_CO	33.7	41.3	24.9

**Fig. 3:**
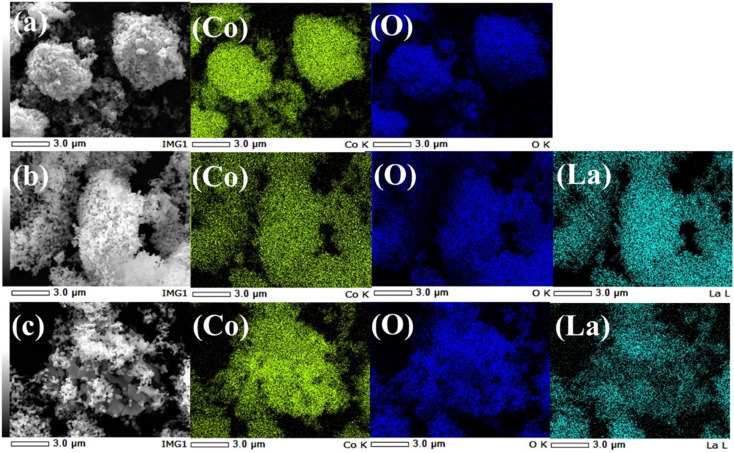
EDS elemental mapping images for the synthesized materials: (a) CO, (b) LCO, and (c) the LCO_CO composite.

To quantitatively support the morphological observations, EDS elemental mapping was performed (Fig. 3 and Table S3). Fig. 3 illustrates the energy dispersive X-ray spectroscopic (EDS) elemental mapping images for the synthesized materials: (a) CO, (b) LCO, and (c) the LCO_CO composite. The maps provide a clear visual correlation with the FE-SEM images in Fig. 2, demonstrating that the constituent elements are precisely localized within the primary particle boundaries. The results unequivocally demonstrate that La, Co, and O are uniformly distributed at the nanoscale, with the quantitative elemental ratios in Table S3 closely matching the theoretical compositions. For the single-phase materials (CO and LCO), the uniform distribution of their respective elements (Co and O for CO; La, Co, and O for LCO) is strictly consistent with the particulate morphology observed in the SEM images, confirming the formation of compositionally homogeneous, single-phase crystalline particles. The uniformity is particularly remarkable in the composite sample, LCO_CO (Fig. 3(c)). The distribution maps for La and Co exhibit a close overlap, which strongly supports the conclusion that the LCO and CO phases are not merely a physical mixture but are homogeneously composited at a fine scale. This high compositional homogeneity observed by EDS in the LCO_CO composite correlates well with the structural features seen in the SEM images (Fig. 2(c)). The increased agglomeration observed in the SEM of LCO_CO is therefore suggested to result from the intimate, nanometer-scale junction and strong interfacial interaction between the LCO and CO phases, rather than simple particle adhesion. In summary, the LCO_CO composite was successfully synthesized as a nanostructure with a homogeneous distribution of La, Co, and O, affirming the successful preparation of a material where the CO and LCO phases are uniformly integrated. This microstructural feature, achieved *via* the coprecipitation method, is crucial for promoting the synergistic effects necessary for superior catalytic performance.

### 3.3 Specific surface area and porosity (BET analysis)

**Fig. 4:**
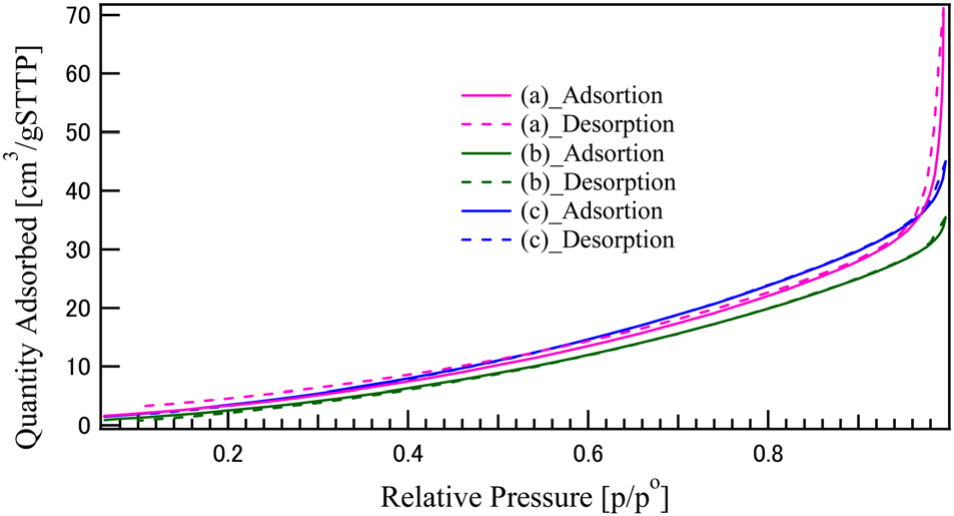
N_2_ adsorption–desorption isotherms of the catalysts synthesized by the coprecipitation method: (a) CO, (b) LCO, and (c) LCO_CO composite.

Fig. 4 shows the nitrogen adsorption–desorption isotherms for the synthesized materials: (a) CO, (b) LCO, and (c) the LCO_CO composite. Table 1 summarizes the specific surface area values calculated from the isotherms using the Brunauer–Emmett–Teller (BET) method. The isotherms for all samples exhibit a hysteresis loop where the adsorption and desorption curves do not coincide, particularly in the high relative pressure region (*P*/*P*_0_ ≥ 0.4). This characteristic adsorption–desorption behavior is classified as Type IV by the IUPAC definition, clearly indicating the presence of mesopores in all materials.^24^ The steep increase in the adsorption amount observed in the high-pressure region (*P*/*P*_0_ ≈ 1.0) likely reflects the existence of macropores (inter-particle voids) formed within the agglomerated structures. As shown in Table 1, the calculated BET specific surface areas for the single components are 20.8 m^2^ g^−1^ for CO (a) and 22.8 m^2^ g^−1^ for LCO (b). The LCO_CO composite (c) yielded the largest specific surface area at 24.6 m^2^ g^−1^. Although the difference in specific surface area across all samples is relatively small, the results are consistent with the SEM observations (Fig. 2), which confirmed that the fundamental particle morphology and size did not undergo substantial changes upon composite formation. The slight but notable increase in the BET specific surface area for the LCO_CO composite (24.6 m^2^ g^−1^) is inferred to be the result of one or both of the following factors:^24,25^ the nanoscale, homogeneous compounding of the LCO and CO phases, as supported by EDS, leads to an increase in the total accessible surface area at the interface between the two phases. The enhanced agglomeration observed in the SEM of the composite may have increased the total volume of inter-particle meso- and macropores. The consistent observation of the IUPAC Type IV hysteresis loop across all samples supports the presence of an accessible porous structure, implying that the CO and LCO particles are not completely closed but possess voids between the agglomerated particles.^24–26^ This porous structure is expected to be beneficial for the subsequent electrochemical evaluation, as it facilitates the penetration of the electrolyte and the diffusion of reactants (oxygen) during the ORR and OER processes.

### 3.4 Analysis of chemical states

The surface elemental compositions and chemical states of the synthesized samples were investigated by XPS. The survey spectra of CO, LCO, and the LCO_CO composite (Fig. S5, SI) confirm the presence of La, Co, and O as the primary constituents, along with C from the reference carbon, with no detectable impurities.

**Fig. 5:**
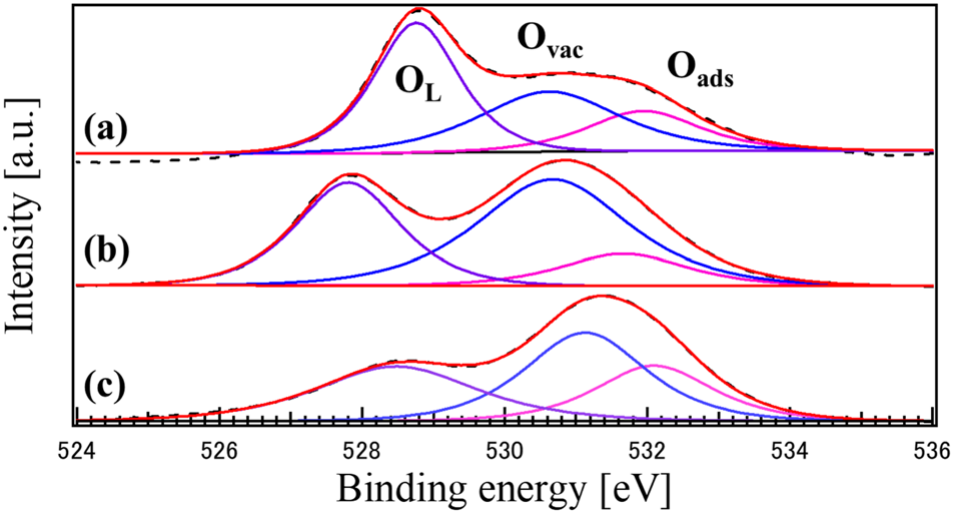
XPS O 1s core-level spectra of the catalysts synthesized by the coprecipitation method: (a) CO, (b) LCO, and (c) LCO_CO composite.

Fig. 5 shows the high-resolution XPS O 1s core-level spectra for CO, LCO, and the LCO_CO composite, with each peak clearly labeled according to its chemical state assignment. The spectra were deconvoluted into three distinct components: lattice oxygen (O_Lattice_) at approximately 529.5 eV, oxygen vacancies or defective oxygen species (O_vac_) at approximately 531.3 eV, and surface-adsorbed species (O_ads_) such as hydroxyl groups or water molecules at binding energies above 532.5 eV.

To confirm the assignment of the O_vac_ peak, Ar-ion sputtering was performed to remove surface-adsorbed contaminants (Fig. S6, SI). The persistence of the peak at 531.3 eV after sputtering confirms that this component originates from inherent structural oxygen vacancies rather than transient surface adsorption. The O_Lattice_ component is assigned to the intrinsic metal–oxygen (La–O and Co–O) bonds within the perovskite and spinel crystal lattices. Notably, the LCO_CO composite exhibits a significantly higher relative area for the O_vac_ peak (41.3%) compared to single-phase CO (35.3%), as detailed in Table 2. This enhancement in oxygen vacancy concentration is attributed to the structural strain and lattice distortion at the hetero-interfaces between the LCO and CO phases, as confirmed by XRD analysis. The increased density of these defective oxygen states modulates the electronic structure of the catalyst surface, which is a key factor in lowering the energy barrier for oxygen intermediate adsorption and thus enhancing the bifunctional catalytic activity.

During the simultaneous growth of these two distinct phases *via* coprecipitation, the crystallographic mismatch induces lattice distortion at the grain boundaries. To compensate for this localized strain and maintain electrical neutrality, the formation energy of oxygen vacancies is reduced, resulting in a higher density of V_o_ at the hetero-interface. As confirmed by SEM and EDS, the LCO and CO phases co-crystallize at the nanoscale, leading to misorientation and disruption of atomic arrangement at the interface. This structural strain makes the lattice oxygen more susceptible to removal, thereby creating oxygen vacancies. The formation of these structural defects is highly relevant, as the presence of oxygen vacancies in mixed metal oxides is widely recognized as a factor that dramatically enhances catalytic activity for the ORR and OER by optimizing the adsorption and electronic properties of the catalyst surface.

**Fig. 6:**
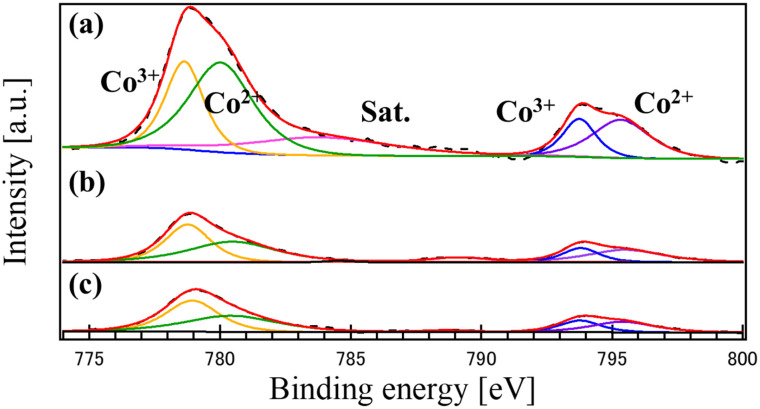
XPS Co 2p core-level spectra of the catalysts synthesized by the coprecipitation method: (a) CO, (b) LCO, and (c) LCO_CO composite.

Fig. 6 shows the XPS Co 2p core-level spectra for the synthesized samples: (a) CO, (b) LCO, and (c) the LCO_CO composite. The Co 2p spectra for all samples clearly exhibit characteristic peaks attributed to both Co^3+^ and Co^2+^ oxidation states.^29^ Upon deconvolution, it was observed that the composite LCO_CO (c) demonstrated the highest peak area ratio of Co^3+^ when compared to the single-phase CO and LCO materials.^27–31^ The Co^3+^ species is generally recognized as being highly effective for enhancing catalytic activity in various reactions.^30–33^ The XPS analysis confirms that the formation of the LCO_CO composite significantly influences the local electronic environment, resulting in a favorable adjustment of the valence balance, specifically increasing the Co^3+^/Co^2+^ ratio (Table 3). This change can be attributed to the interfacial effects arising from the intimate coupling of the LCO (perovskite) and CO (spinel) phases. The intimate contact between LaCoO_3_ and Co_3_O_4_ induces an interfacial electronic effect, as observed in similar hetero-junction systems.^34^ At the interface where the two phases are joined at the nanoscale (as evidenced by SEM and EDS), the local electronic state is perturbed, leading to a redistribution of charge and stabilization of a higher Co^3+^ concentration than found in the isolated phases. The observed increase in the Co^3+^ ratio in the LCO_CO composite is considered a critical factor that will directly contribute to the anticipated enhancement of its catalytic performance towards the ORR and OER. This finding, along with the evidence for oxygen vacancy formation and lattice strain, strongly supports the hypothesis that the coprecipitation method successfully induced beneficial synergistic structural and electronic effects in the composite material.^28–31^

**Table 3:** Relative peak area ratios of Co 2p components (Co^2+^ and Co^3+^) derived from XPS deconvolution for CO, LCO, and LCO_CO composite samples

Sample	Co^2+^ [%]	Co^3+^ [%]
CO	63.0	37.0
LCO	55.1	44.9
LCO_CO	50.6	49.4

### 3.5 Electrochemically active surface area (ECSA) analysis

**Fig. 7:**
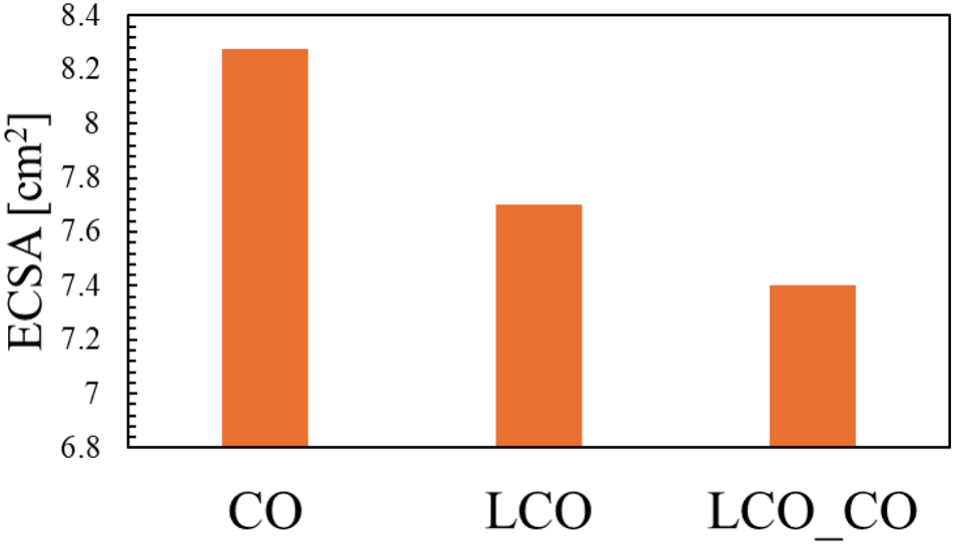
Electrochemically active surface area (ECSA) of CO, LCO, and LCO_CO composite determined from the double-layer capacitance (*C*_dl_) measurements.

Fig. 7 shows the ECSA values calculated from the cyclic voltammetry (CV) measurements (Fig. S7, SI). ECSA serves as a critical metric reflecting the true contact area between the electrode and the electrolyte within the catalyst layer, and its value is essential for evaluating the intrinsic activity of the catalyst. Contrary to the BET specific surface area results (Table 1), where the order was LCO_CO > LCO > CO (24.6 m^2^ g^−1^), the ECSA values followed the sequence: CO > LCO > LCO_CO. The fact that the LCO_CO composite, which exhibited the highest physical surface area, yielded a lower ECSA suggests a significant discrepancy between the physically accessible surface area and the electrochemically active surface area. This divergence is primarily attributed to the differing nature of the two measurements. BET measurement can evaluate the total surface area of the dry powder using small N_2_ gas molecules, while ECSA measurement strongly depends on the wettability of the catalyst in the liquid electrolyte and the effective pore volume accessible to the electrolyte ions (KOH aqueous solution). The single-phase CO sample achieved the largest apparent ECSA, suggesting that its surface structure and agglomeration state allowed for the most effective formation of the electrode/electrolyte interface in the liquid medium. The LCO_CO composite's lower ECSA, despite its high BET value, is potentially linked to its pore structure. This is supported by the pore size distribution (PSD) analysis (Fig. S8, SI), which reveals that the LCO_CO composite contains a higher fraction of smaller mesopores compared to the single-phase CO and LCO. The hysteresis loop shape observed in the BET isotherm (Fig. 4) further suggests that the pores—either inter-particle voids or internal pores—within the LCO_CO composite may have narrower necks (entrances) compared to the single materials. While these narrow pores permit the adsorption of small gas molecules (N_2_), they are likely to increase the steric hindrance and mass transport resistance for the diffusion of solvated electrolyte ions (KOH aqueous solution). To rule out extrinsic factors, it should be noted that a constant binder concentration was used for all samples, and the CV measurements were conducted within a strictly non-faradaic region to prevent electrolyte decomposition. Thus, the observed discrepancy primarily arises from the limited accessibility of the liquid electrolyte to the narrow-neck pores of the composite. Consequently, a portion of the composite's high surface area becomes inaccessible to the electrolyte, leading to a reduction in the electrochemically active surface area. Therefore, the final ECSA analysis strongly suggests that the superior ORR and OER activity demonstrated by the LCO_CO composite is not primarily governed by its slightly higher physical surface area, but rather by the enhancement of its intrinsic activity at the hetero-interfaces. To further investigate the intrinsic catalytic activity independent of the surface area, the OER and ORR polarization curves were normalized by the calculated ECSA (see Fig. S9 and S10, SI). The *C*_dl_ values and the details of the ECSA calculation process are summarized in Tables S4 and S2, respectively. Notably, even after ECSA normalization, the LCO_CO composite maintained the highest specific current density among all synthesized catalysts. This result strongly suggests that the superior bifunctional activity demonstrated by the LCO_CO composite is not primarily governed by its physical surface area, but rather by the enhancement of its intrinsic activity. This phenomenon, where high catalytic performance is achieved despite a low electrochemically active surface area, is attributed to the significant leap in intrinsic activity and has been similarly reported for other high-performance catalysts.^35^ This intrinsic enhancement is driven by favorable electronic changes, specifically the increase in the concentration of catalytically active Co^3+^ species and the formation of oxygen vacancies, which are induced by homogeneous nanoscale compounding.

### 3.6 OER and ORR performance

**Fig. 8:**
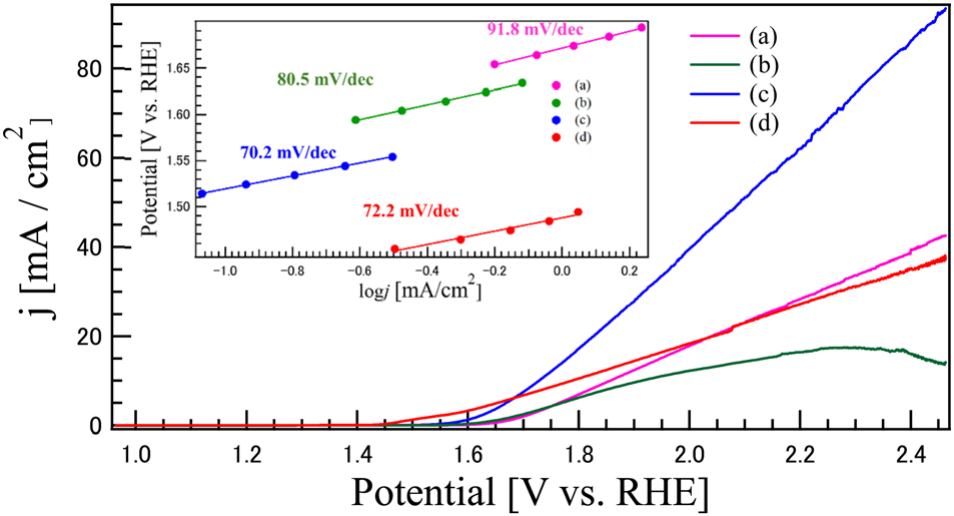
LSV results (in 0.1 M KOH aqueous solution) investigating the OER catalytic properties of the catalysts synthesized by coprecipitation method: (a) CO, (b) LCO, (c) LCO_CO composite, and (d) commercial RuO_2_ for comparison. The inset shows Tafel slopes obtained by the LSV curves.

Fig. 8 presents the linear sweep voltammetry (LSV) results investigating the OER catalytic properties of the synthesized catalysts: (a) CO, (b) LCO, (c) LCO_CO composite, and (d) commercial RuO_2_ for comparison. In all samples except for RuO_2_ (d), a discernible increase in current density was observed near 1.6 V, confirming the onset of the OER. Key OER performance metrics calculated from the LSV curves are summarized in Table 4. The onset potential, a measure of catalytic ease, decreased in the order of RuO_2_ < LCO_CO < LCO < CO. Interestingly, while the A-site cation oxide La_2_O_3_ is known to be electrochemically inactive for the OER, exhibiting an overpotential well exceeding 600 mV to reach 10 mA cm^−2^,^36^ its integration into the perovskite structure (LaCoO_3_) or the composite system significantly enhances the overall activity. The LCO_CO composite exhibited the lowest onset potential among the metal oxide samples at 1.593 V. This metric, critical potential at 10 mA cm^−2^ (*E*_*j*=10_) for practical performance, was significantly lower for LCO_CO at 1.729 V. This value is lower than the high-performance commercial catalyst RuO_2_ (1.788 V) and substantially superior to the single components (CO: 1.857 V; LCO: 1.914 V). This synergistic enhancement suggests that the presence of La effectively modulates the local chemical environment and electronic structure of the Co active sites, optimizing the adsorption energies of OER intermediates, as suggested by the role of A-site cations in similar perovskite systems.^36^ Regarding Tafel Slope, the LCO_CO composite displayed the fastest charge transfer kinetics in the OER rate-determining step, boasting the lowest Tafel slope of 70.2 mV dec^−1^, slightly surpassing that of RuO_2_ (72.2 mV dec^−1^).

**Table 4:** Onset potential, potential at 10 mA cm^−2^, and Tafel slope values for the OER derived from LSV measurements for each sample

Sample	Onset potential (*E*_onset_) V	Potentials at 10 mA cm^−2^	Tafel slope [mV dec^−1^]
(a) CO	1.672	1.857	91.8
(b) LCO	1.647	1.914	80.5
(c) LCO_CO	1.593	1.729	70.2
(d) RuO_2_	1.489	1.788	72.2

The LSV results unequivocally demonstrate that the LCO_CO composite drastically outperforms its single-component counterparts in all OER metrics, achieving an OER activity comparable to benchmark RuO_2_. This dramatic performance enhancement is attributed to the synergistic combination of structural and electronic factors induced by the homogeneous nanoscale compounding. The LCO_CO composite exhibited a peak attributed to oxygen vacancies (V_o_) in the XPS O 1s spectra (Fig. 5). Oxygen vacancies effectively tune the adsorption strength of oxygenated intermediates, thereby lowering the overpotential.^37^ These defect structures, induced by the lattice strain at the mismatched phase interface, are known to act as highly effective catalytic active sites for the OER. The XPS Co 2p spectra showed that LCO_CO possessed the highest ratio of Co^3+^, an oxidation state crucial for enhancing OER activity.^38^ The presence of Co^2+^/Co^3+^ redox couples is known to be beneficial for bifunctional oxygen electrocatalysis.^38^ This electronic optimization was achieved through the localized changes in valence balance at the LCO/CO interface. Specifically, the oxygen vacancies formed at the hetero-interface between LCO and CO are expected to optimize the adsorption energy of oxygen intermediates, thereby effectively suppressing the overpotentials for OER.^39,40^ This interpretation is consistent with recent reports on interface engineering in Co-based oxides, which highlight the synergy between electronic modulation and defect density.^41^ The BET analysis (Table 1) revealed that the LCO_CO composite possessed a slightly higher specific surface area (24.6 m^2^ g^−1^) compared to the single phases. This minor increase is a result of the formation of an efficient meso- and macroporous network between the uniformly compounded nanoparticles (confirmed by SEM and EDS). This enhanced porosity improves electrolyte penetration and reduces mass transport resistance for reactants (*e.g.*, OH^−^), thereby effectively boosting performance, particularly at high current densities (*E*_*j*=10_). The change in the activity sequence between low potential (onset potential: RuO_2_ > LCO_CO > LCO > CO) and high current density (*E*_*j*=10_: LCO_CO > RuO_2_ > CO > LCO) suggests a shift in the rate-determining step. While RuO_2_ and LCO may initially be governed by rapid OH adsorption/desorption kinetics,^25^ the LCO_CO composite, with its combination of enhanced active sites (oxygen vacancies) and improved mass transport (porous structure), maintained a fast charge transfer rate (Tafel slope 70.2 mV dec^−1^) even at high current densities, contributing significantly to its superior performance at 10 mA cm^−2^. A crucial observation is that the LCO_CO composite, despite achieving the highest catalytic performance, recorded the lowest ECSA value. This strongly suggests that the catalytic activity is not simply proportional to the macroscopic surface area quantified by ECSA. Instead, the performance is overwhelmingly dominated by qualitative factors such as the structure and electronic state of the atomic-level active sites (*i.e.*, Co^3+^ enrichment and oxygen vacancy formation), which are not captured by the ECSA measurement. The observation of superior activity notwithstanding a lower ECSA is a direct consequence of enhanced intrinsic activity, a trend consistent with previous studies on advanced electrocatalysts.^35^ This emphasizes the critical importance of optimizing the intrinsic activity of the composite material for efficient OER catalysis.^42–44^

**Fig. 9:**
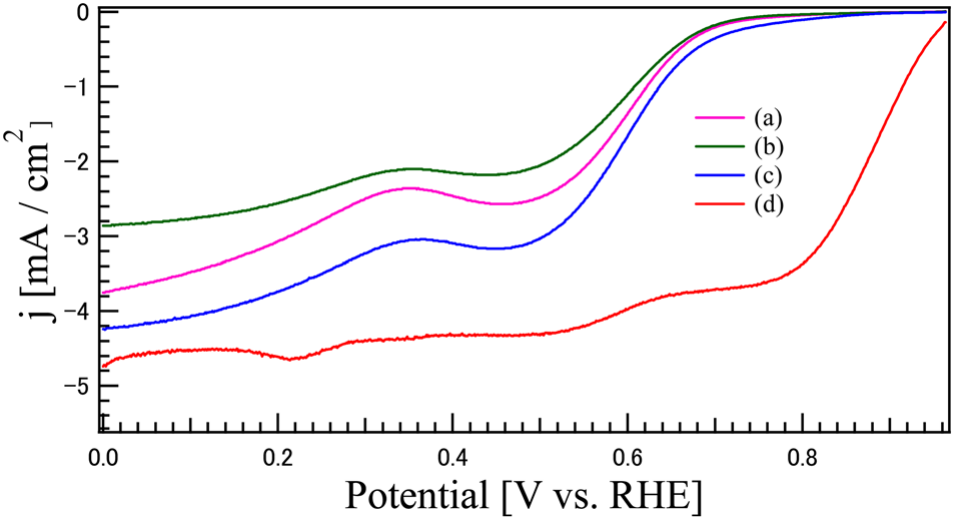
LSV curves (in 0.1 M KOH aqueous solution) for the oxygen reduction reaction (ORR) of the catalysts synthesized by coprecipitation method: (a) CO, (b) LCO, (c) LCO_CO composite, and (d) commercial 20 wt% Pt/C for comparison.

Fig. 9 presents the LSV curves for the oxygen reduction reaction (ORR) of the synthesized catalysts: (a) CO, (b) LCO, (c) LCO_CO composite, and (d) commercial 20 wt% Pt/C for comparison. The ORR performance metrics, including the onset potential (*E*_onset_) and half-wave potential (*E*_1/2_), are summarized in Table 5. While Pt/C (d) demonstrated the highest overall activity, the LCO_CO composite (c) exhibited the most superior performance among the metal oxide catalysts, showing the highest *E*_onset_ (0.686 V) and *E*_1/2_ (0.582 V). The overall performance trend was Pt/C > LCO_CO > CO > LCO, confirming the substantial performance enhancement achieved through the compounding process, similar to the OER results. The wave-like shape of the LSV curves observed for the metal oxide catalysts, such as LCO and CO, suggests the coexistence of both the two-electron reduction pathway (O_2_*via* H_2_O_2_) and the direct four-electron reduction pathway (O_2_ to OH^−^). Given the inherently lower electrical conductivity of metal oxide catalysts, electron transfer often becomes the rate-limiting step, leading to an initial dominance of the two-electron reduction process, followed by a transition to the four-electron process as the potential decreases. This transition is often associated with the observed change in the reaction rate. The improved ORR activity of the LCO_CO composite is primarily attributed to the structural and electronic modifications induced by the homogeneous compounding of LCO and CO, consistent with the mechanism proposed for the OER. Specifically, the oxygen vacancies formed at the hetero-interface between LCO and CO are expected to optimize the adsorption energy of oxygen intermediates, thereby effectively suppressing the overpotentials for ORR.^39,40^ This interpretation is consistent with recent reports on interface engineering in Co-based oxides, which highlight the synergy between electronic modulation and defect density. The XPS analysis (Fig. 6) demonstrated an increased concentration of Co^3+^ species in the LCO_CO composite. Co^3+^ is known to be effective at accepting electrons from O_2_ molecules or OH^−^ intermediates, thereby promoting the intermediate adsorption/desorption processes that limit the ORR kinetics and enhancing catalytic activity.^44–47^ The oxygen-deficient structure identified in the O 1s spectra (Fig. 5) is believed to function as an additional type of active site that facilitates the adsorption and activation of the O_2_ molecule. The LCO_CO composite exhibited a slightly higher specific surface area (24.6 m^2^ g^−1^) and an efficient mesoporous structure (Fig. 4) compared to the single phases. This microstructural advantage, resulting from the nanoscale compounding (SEM and EDS), is hypothesized to maximize the electrode/electrolyte interfacial area and promote the diffusion of O_2_ into the catalyst layer, contributing to performance gains, particularly at higher current densities (*E*_onset_ and *E*_1/2_). The higher *E*_onset_ and *E*_1/2_ values of the LCO_CO composite compared to CO and LCO suggest the formation of catalytic sites that favor the more efficient four-electron reduction pathway. This is likely due to the optimized Co^3+^ electronic state promoting the scission of the O–O bond in the O_2_ molecule, suppressing H_2_O_2_ generation, and channeling the reaction directly toward OH^−^ production (four-electron pathway).^42–44^

**Table 5:** Onset potential, half-wave potential, electron transfer number, and H_2_O_2_ formation rate values for the ORR derived from LSV measurements for each sample. The onset potential was determined at a current density of 0.5 mA cm^−2^

Sample	Onset potential (*E*_onset_) [V]	Half-wave potential (*E*_1/2_) [V]	Electron transfer numbers (*n*)@0.464 V	Formation rate of H_2_O_2_@0.464 V [%]
(a) CO	0.659	0.567	3.39	31.4
(b) LCO	0.651	0.573	3.61	19.9
(c) LCO_CO	0.686	0.582	3.73	13.4
(d) Pt/C	0.939	0.864	3.71	14.4

To further assess the practical potential of the LCO_CO composite, its bifunctional catalytic activity was compared with recently reported state-of-the-art catalysts (Table S5). The potential difference (Δ*E*) between the *E*_1/2_ for ORR and the *E*_*j*=10_ for OER, which serves as a metric for overall bifunctionality, was determined to be 1.14 V. This value is highly competitive with other recent perovskite-based and transition metal oxide systems, confirming that the synergistic interface between LaCoO_3_ and Co_3_O_4_ effectively promotes both oxygen reduction and evolution reactions.

**Fig. 10:**
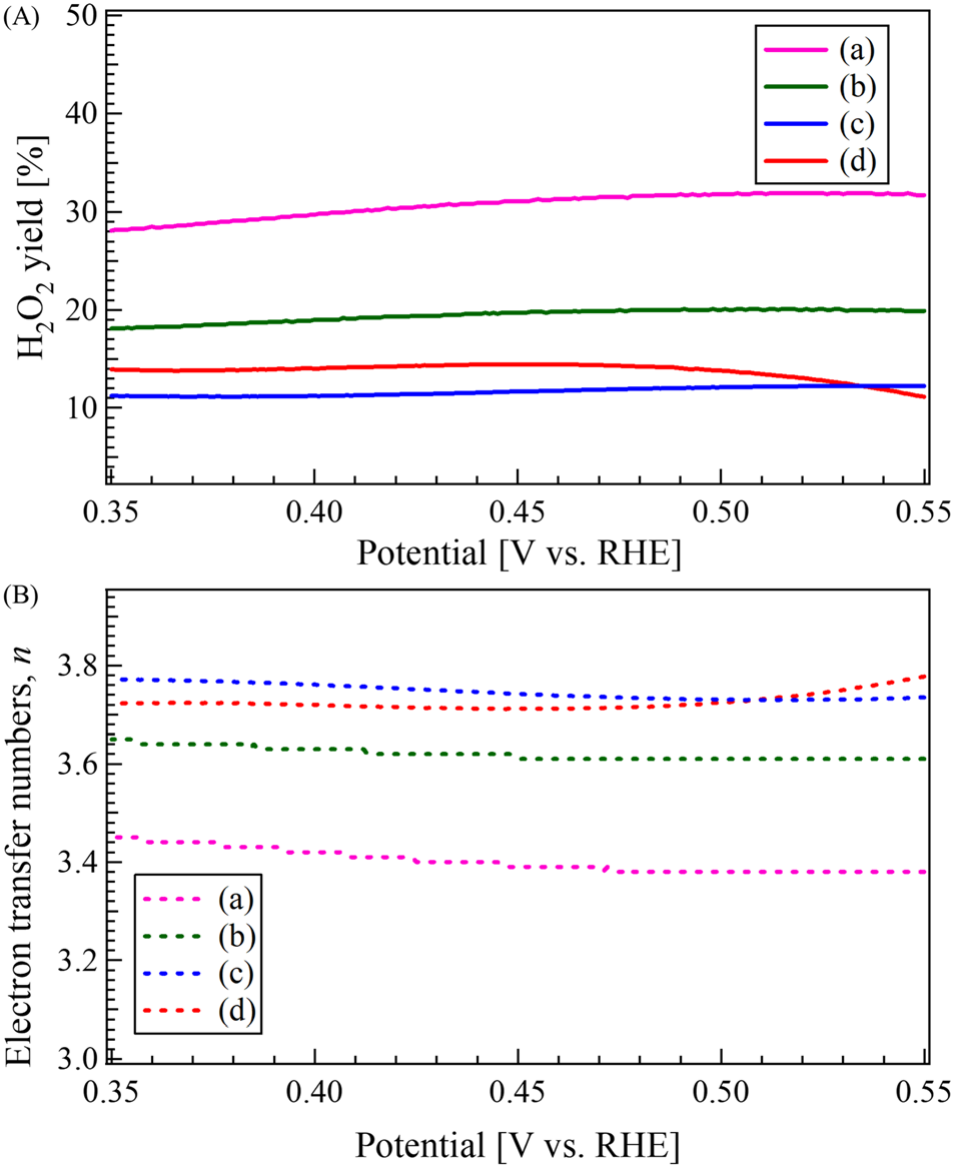
(A) Calculated H_2_O_2_ yield (H_2_O_2_ yield [%]) and (B) electron transfer numbers (*n*) as a function of potential, derived from LSV measurements for the (a) CO, (b) LCO, (c) LCO_CO catalysts synthesized by the coprecipitation method and (d) commercial Pt/C catalyst.

Fig. 10 presents (A) the calculated H_2_O_2_ yield and (B) the electron transfer number (*n*) as a function of potential, derived from the LSV measurements for (a) CO, (b) LCO, (c) LCO_CO, and (d) Pt/C. This analysis confirms that the LCO_CO composite possesses a higher selectivity toward the four-electron pathway compared to the single-component oxides. At the potential of 0.464 V, the LCO_CO composite (c) achieved an electron transfer number of *n* = 3.73 and a H_2_O_2_ yield of 13.4%, demonstrating superior four-electron selectivity compared to CO (*n* = 3.39, 31.4% yield) and LCO (*n* = 3.61, 19.9% yield). In the more negative potential region (kinetic region), LCO_CO showed a slightly more dominant four-electron reaction compared to Pt/C. This indicates that the Co^3+^ active sites in LCO_CO contribute to lowering the activation energy of the elementary reaction steps more effectively than Pt/C in this specific potential range. However, the final limiting current density of LCO_CO was lower than that of Pt/C, presumably due to the smaller total number of accessible active sites compared to the highly utilized Pt/C catalyst surface. This evidence of high selectivity towards the four-electron reduction pathway further supports the conclusion that the nanoscale composite structure successfully optimized the electronic state and defect chemistry of Co, resulting in enhanced intrinsic catalytic activity for ORR, which is a prerequisite for high-performance Li–O_2_ batteries.

**Fig. 11:**
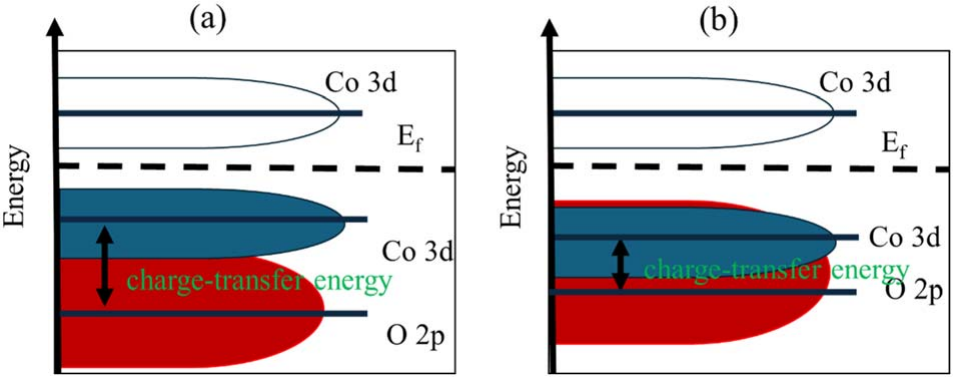
Energy relationship diagrams of Co 3d and O 2p orbitals in the DOS: (a) case of Co^2+^, (b) case of Co^3+^.

Fig. 11 shows the conceptual correlation diagram of the Co 3d and O 2p orbitals, constructed based on the theoretical models reported in recent literature,^48,49^ which act as the governing factors of this mechanism. Specifically, the structural strain at the LCO/CO interface, as confirmed by XRD (Fig. 1), promotes the formation of a high concentration of oxygen vacancies (41.3% from Table 2). As Co^3+^ is generated, the energy level of the Co 3d orbital decreases; concurrently, the introduction of these oxygen vacancies raises the energy level (band center) of the O 2p orbital, thereby narrowing the energy gap between the two (Fig. 11(b)). As a result, the Co 3d and O 2p orbitals strongly hybridize, leading to a significant decrease in the charge transfer energy.^48,49^

**Fig. 12:**
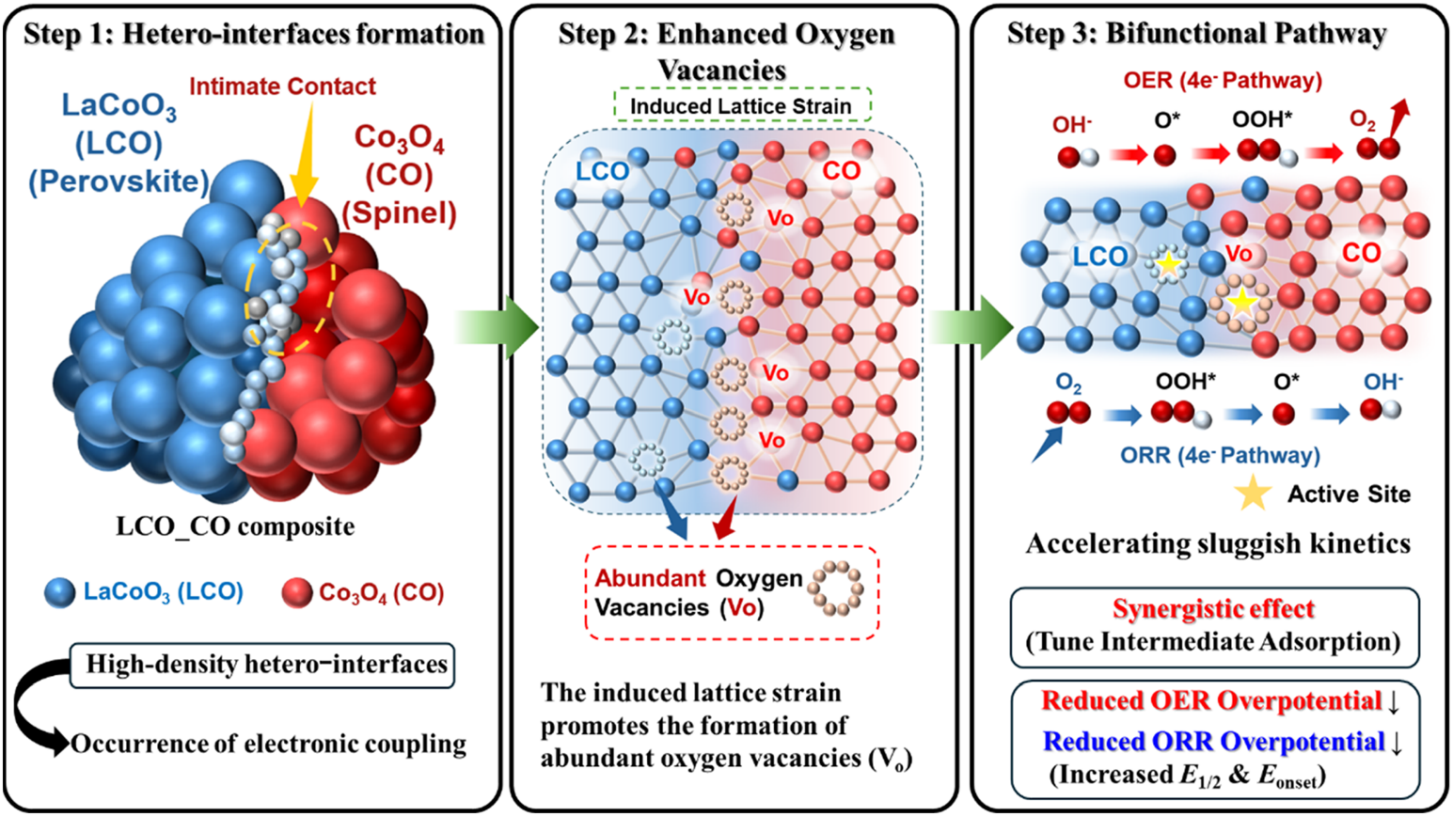
Schematic illustration of the synergistic mechanism in LCO_CO composite. Step 1: formation of high-density hetero-interfaces. Step 2: strain-induced oxygen vacancy formation. Step 3: bifunctional ORR/OER pathways *via* optimized electronic states at the interface.

The proposed synergistic mechanism of the LCO_CO composite is clearly depicted in the schematic illustration (Fig. 12). As shown in the scheme, the intimate nanoscale contact between the perovskite (LCO) and spinel (CO) phases creates a high density of hetero-interfaces where electronic coupling occurs. The induced lattice strain at these mismatched boundaries promotes the formation of abundant oxygen vacancies (V_o_), which act as highly effective catalytic active sites. The diagram explicitly illustrates how this synergistic interface optimizes the adsorption/desorption energies of the OH*, O*, and OOH* intermediates through a unified bifunctional pathway, thereby accelerating sluggish kinetics and reducing overpotentials for both ORR and OER.

## 4 Conclusions

In this study, we successfully synthesized the composite metal oxide LaCoO_3__Co_3_O_4_ (LCO_CO) along with the single components (LCO and CO) using the coprecipitation method and evaluated their bifunctional catalytic activity (OER and ORR) for application as air-cathode catalysts in Li–O_2_ batteries. The LCO_CO composite exhibited the most superior bifunctional catalytic activity among all synthesized materials, achieving the lowest overall overpotential (Δ*E* = 1.14 V) for the ORR/OER cycle. This excellent performance is attributed to the high ORR activity and the corresponding remarkable improvement in OER activity, where the *E*_*j*=10_ value (1.729 V) was superior to commercial RuO_2_ (1.788 V). Crucially, the LCO_CO composite demonstrated the highest catalytic performance despite possessing the lowest electrochemically active surface area (ECSA). This finding strongly suggests that the overall catalytic activity is not limited by the macroscopic surface area (quantity), but rather by qualitative factors such as the structure and electronic state of the atomic-level active sites (*i.e.*, intrinsic activity). Furthermore, the XRD and XPS analyses provided strong evidence that the introduction of Co^2+^ ions during the composite synthesis process induced lattice defects in the La sites. This structural modification led to a significant increase in oxygen vacancies within the lattice and an optimization of the Co^3+^/Co^2+^ ratio. Moreover, electronic structure analysis revealed that these modifications lowered the charge transfer energy by narrowing the gap between the Co 3d and O 2p band centers. This electronic modulation effectively promotes the participation of lattice oxygen *via* the Lattice Oxygen Mechanism (LOM) and optimizes the adsorption strength of oxygenated intermediates. These synergistic effects at the hetero-interface, combining structural strain and electronic coupling, are concluded to be the dominant factors that facilitate electron transfer during both the OER and ORR steps, ultimately contributing to the excellent bifunctional catalytic performance observed in the LaCoO_3__Co_3_O_4_ composite.

## Author contributions

Conceptualization and methodology, T. I.; experimental and data analysis, A. S., H. T., Y. F., T. I., T. I., K. Y., and G. P.; writing—original draft preparation, A. S. and T. I.; writing—review and editing, T. I.; supervision, T. I.; project administration and funding acquisition, T. I. All authors have read and agreed to the published version of the manuscript.

## Conflicts of interest

There are no conflicts to declare.

## Supplementary Material

RA-016-D5RA09353H-s001

## Data Availability

The data supporting this article have been included as part of the supplementary information (SI). Supplementary information: optimization of LCO : CO molar ratio, determination of ECSA and specific activity, Rietveld refinement patterns of the LCO_CO composite, STEM images, XPS spectra, pore size distribution, current density normalized by ECSA of LSV curves, and comparison of bifunctional catalytic activities (ORR and OER). See DOI: https://doi.org/10.1039/d5ra09353h.
