# Supplementary material for: Synergistic LaCoO3@Co3O4 bifunctional catalyst for efficient oxygen evolution and reduction: achieving low polarization
Source: RSC Adv. 2026 Apr 22;16(23):20616–28. doi: 10.1039/d5ra09353h (PMC13101756; doi:10.1039/d5ra09353h)
Supplement: RA-016-D5RA09353H-s001 [file RA-016-D5RA09353H-s001.pdf]

Supplementary Information (SI) for RSC Advances.
This journal is © The Royal Society of Chemistry 2026

Supporting Information

Synergistic $LaCoO_3$@Co_3O_4 Bifunctional Catalyst for Efficient Oxygen Evolution and Reduction: Achieving Low Polarization

Akihito Shio[a], Hayato Takada[a], Yuna Fujiwara[a], Taketo Imamur[a], Toshiki Iwato[a], Kouki Yamamoto[a], Gasidit Panomsuwan[b], Takahiro Ishizaki[c],*

[a]Materials Science and Engineering, Graduate School of Engineering and Science, Shibaura Institute of Technology, Tokyo 135-8548, Japan
[b]Department of Materials Engineering, Faculty of Engineering, Kasetsart University, Bangkok 10900, Thailand
[c]College of Engineering, Shibaura Institute of Technology, Tokyo 135-8548, Japan.

* E-mail：ishizaki@shibaura-it.ac.jp

S1. Optimization of LCO : CO Molar Ratio

To determine the optimal composition, we synthesized composites with different molar ratios of LCO and CO (LCO : CO = 1:1, 1:3, and 1:5) and evaluated their bifunctional activities.

S2. Determination of ECSA and Specific Activity

The ECSA was estimated from the double-layer capacitance (C_{dl}) determined by CV measurements at various scan rates in the non-faradaic region. A specific capacitance (C_s) of 0.04 mF cm^{-2} was assumed for the calculation (ECSA = C_{dl} / C_s). The specific activity was then obtained by normalizing the measured current by the ECSA.

$$\mathrm{ECSA} = \frac{C_{dl}}{C_s}$$

$$i = C_{dl} \cdot v \quad (v\text{: scan rate [mV/s]})$$

$$i = \frac{(j_{cathode@0.25V} - j_{anode@0.25\,V})}{2}$$

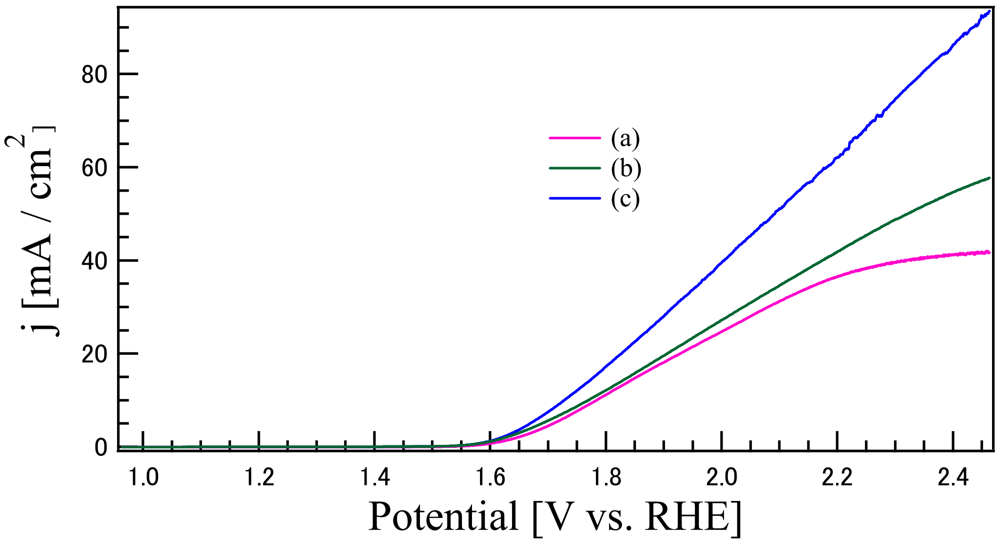

Fig. S1. LSV results (in 0.1M KOH aqueous solution) investigating the OER catalytic properties of the catalysts synthesized by the coprecipitation method: (a) LCO_CO_1, (b) LCO_CO_5, (c) LCO_CO composite. (LCO_CO_1 : La : Co = 1 : 1 (molar ratio), LCO_CO_5 : La : Co = 1 : 5 (molar ratio), LCO_CO : La : Co = 1 : 3 (molar ratio)).

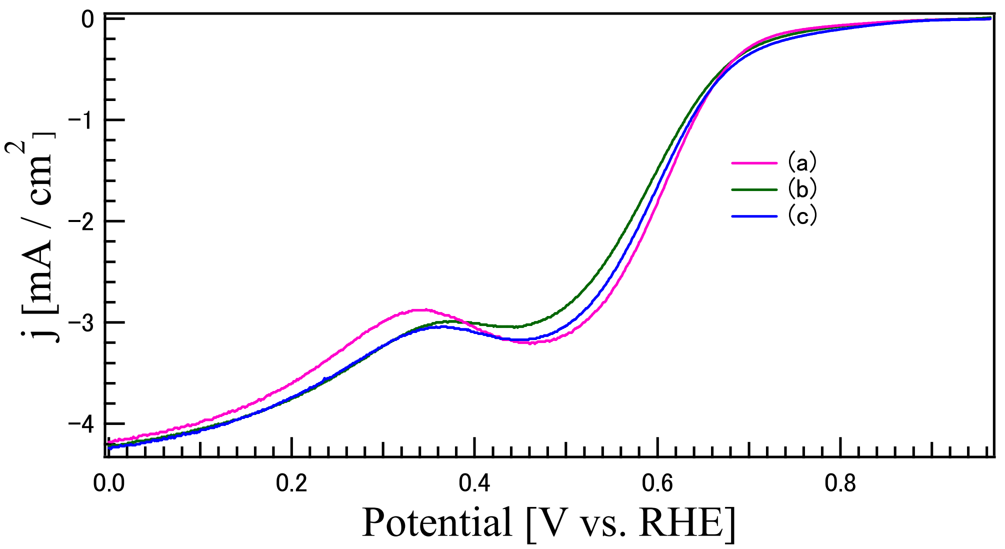

Fig. S2. LSV curves (in 0.1M KOH aqueous solution) for the oxygen reduction reaction (ORR) of the catalysts synthesized by the coprecipitation method: (a) LCO_CO_1, (b) LCO_CO_5, (c) LCO_CO composite. (LCO_CO_1 : La : Co = 1 : 1 (molar ratio), LCO_CO_5 : La : Co = 1 : 5 (molar ratio), LCO_CO : La : Co = 1 : 3 (molar ratio)).

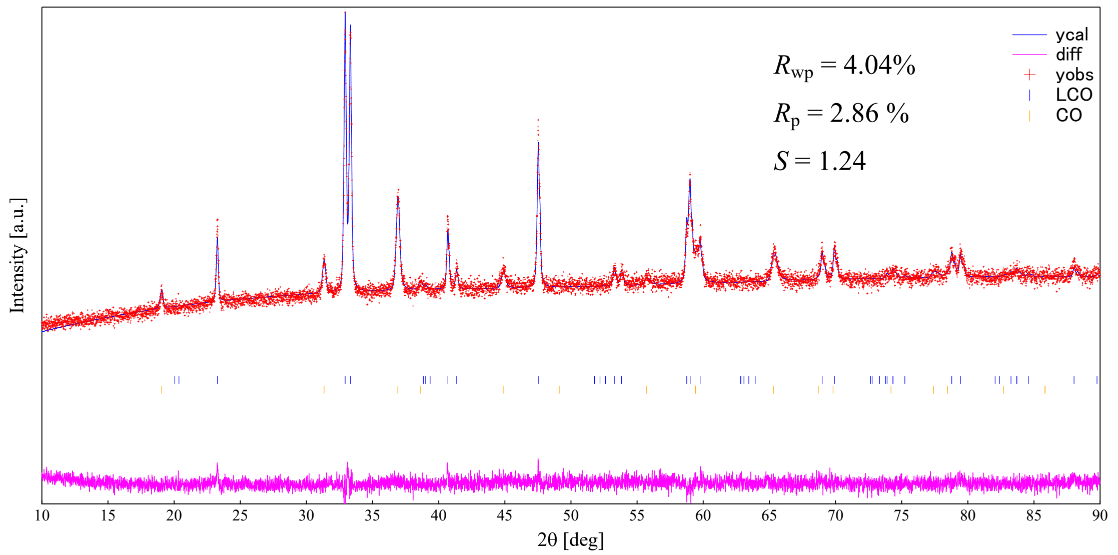

Fig. S3. Rietveld refinement patterns of the LCO_CO composite. The observed intensity is represented by red crosses (y_{obs}), the calculated pattern by a blue line (y_{cal}), and the difference between them by a magenta line (diff) at the bottom. The vertical tick marks indicate the Bragg reflection positions for $LaCoO_3$ (blue, upper) and Co_3O_4 (orange, lower). The refinement was performed using the WPPF method, yielding reliable fitting parameters: R_{wp} = 4.04%, R_p = 2.86%, and S = 1.24.

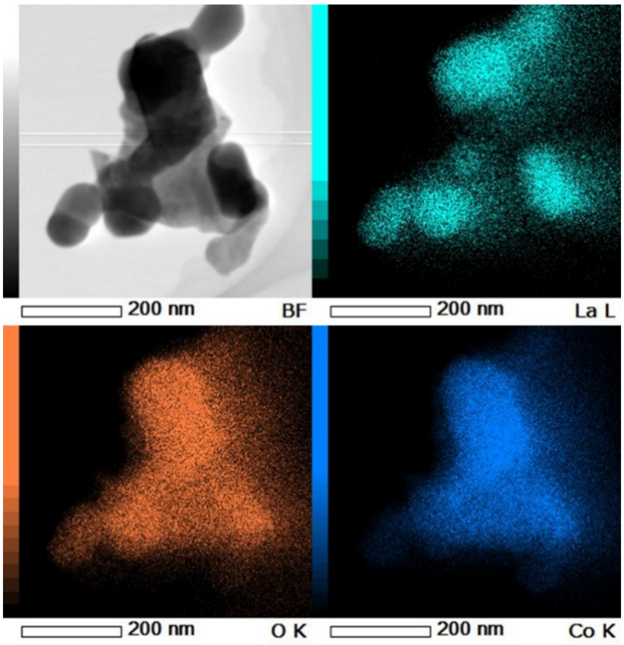

Fig. S4. (a) STEM-BF image and (b-d) corresponding EDS elemental mapping images (La, O, and Co) of the LCO_CO composite. The scale bars represent 200 nm.

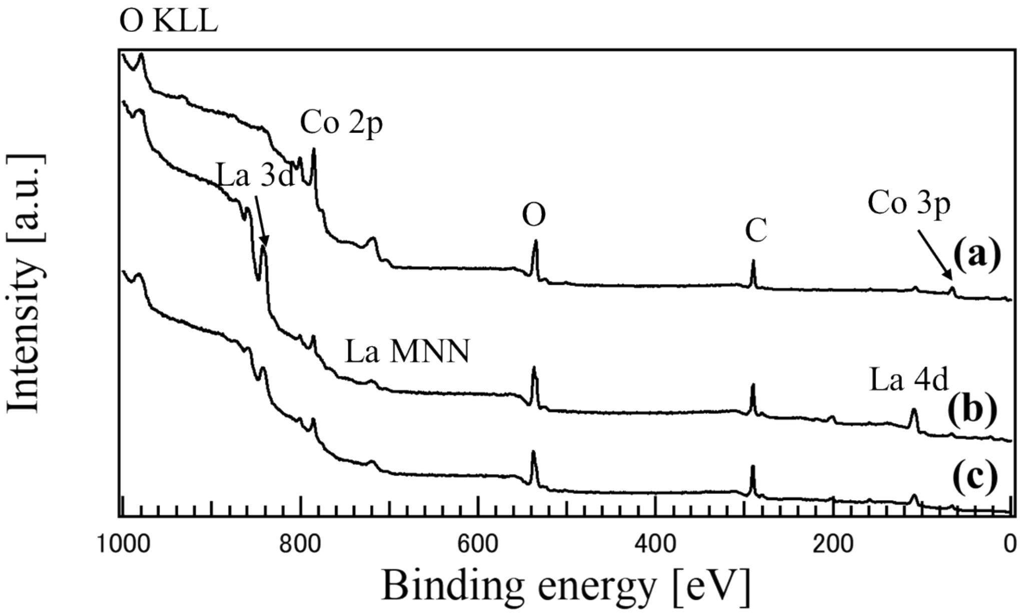

Fig. S5. XPS survey spectra of (a) CO, (b) LCO, and (c) LCO_CO composite, showing the characteristic peaks of La, Co, O, and C.

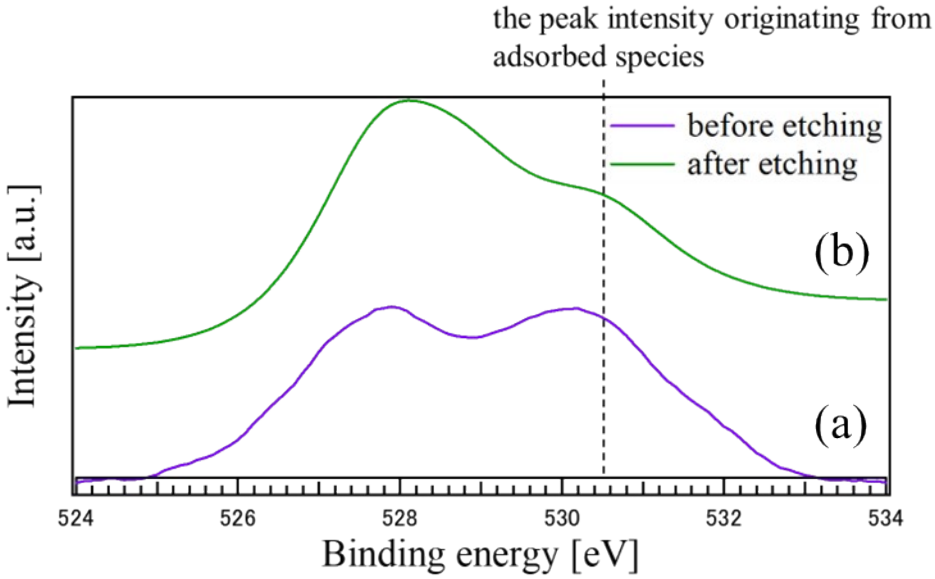

Fig. S6. XPS O 1*s* spectra of the LCO_CO composite (a) before and (b) after Ar-ion sputtering for 5 seconds. The persistence of the peak at ~531.3 eV after the removal of surface contaminants confirms its assignment to lattice oxygen vacancies (O_{vac}) rather than purely adsorbed species.

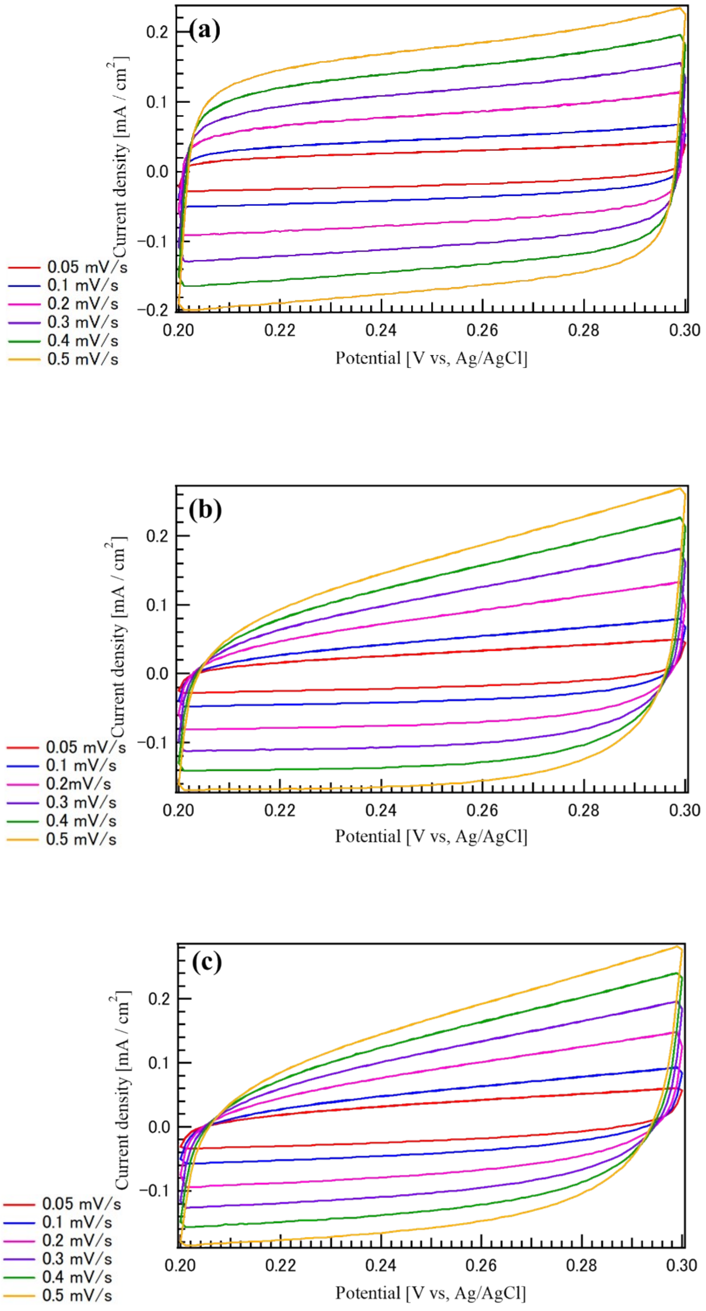

Fig.S7. CV curves (in 1 M KOH) of the catalysts synthesized by the coprecipitation method: (a) CO, (b) LCO, and (c) LCO_CO.

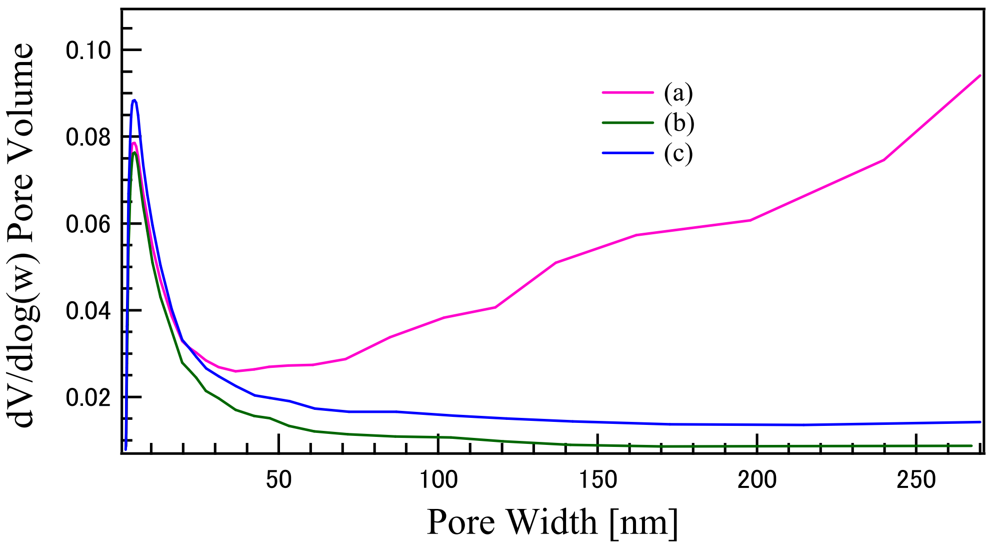

Fig. S8. Pore size distribution (PSD) curves of (a) CO, (b) LCO, and (c) LCO_CO composite calculated from the N_2 adsorption isotherms using the BJH method.

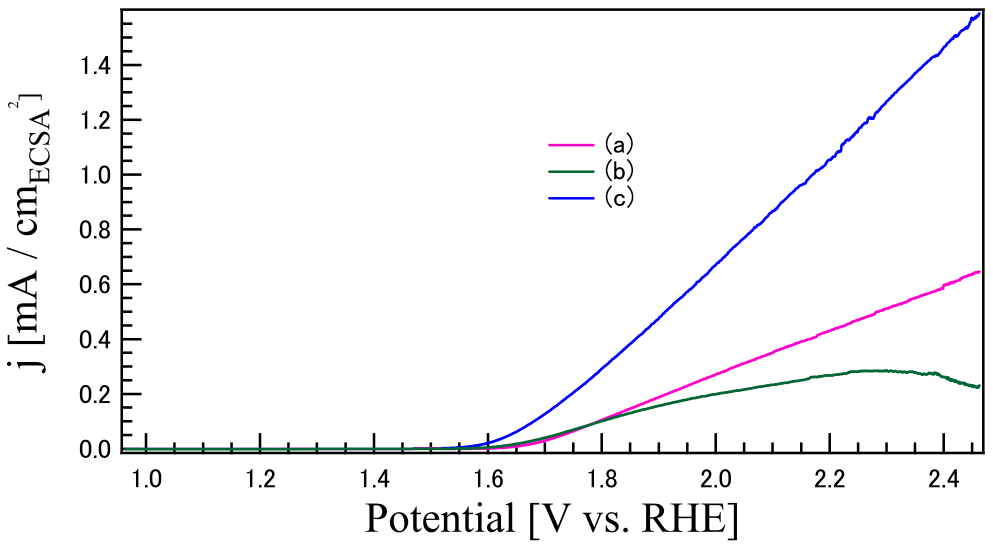

Fig. S9. Current density normalized by ECSA of LSV curves (in 0.1M KOH aqueous solution) for the oxygen evolution reaction (OER) of the catalysts synthesized by the coprecipitation method: (a) CO, (b) LCO, and (c) LCO_CO composite.

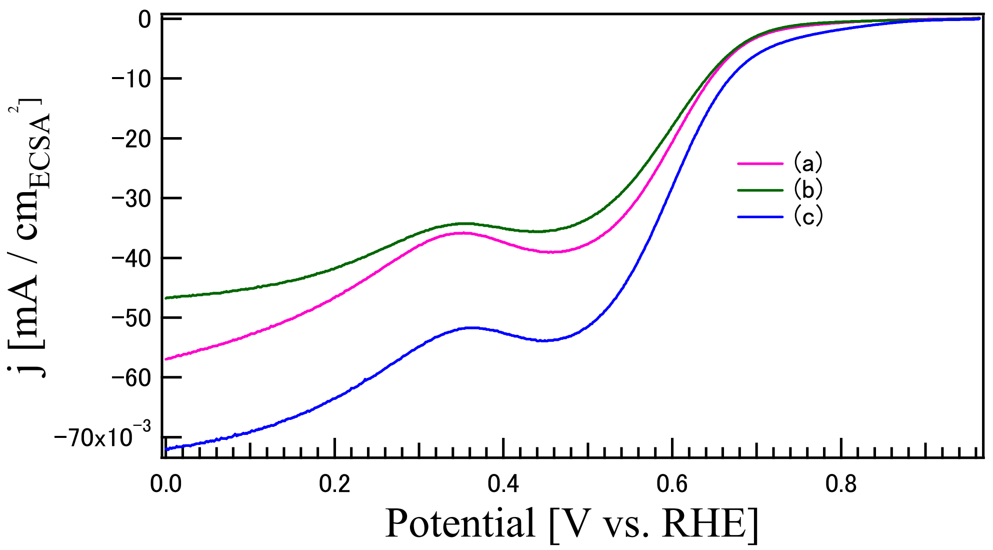

Fig. S10. Current density normalized by ECSA of LSV curves (in 0.1M KOH aqueous solution) for the oxygen reduction reaction (ORR) of the catalysts synthesized by the coprecipitation method: (a) CO, (b) LCO, and (c) LCO_CO composite.

Table S1. Comparison of ORR and OER catalytic activity parameters for LCO_CO composites synthesized with different LCO : CO molar ratios.

Sample	Half-wave potential ($E_{1/2}$) [V]	Potentials at 10 mA/cm^2 [V]	ΔE [V]
(a) LCO_CO_1	0.585	1.785	1.2
(b) LCO_CO_5	0.561	1.770	1.21
(c) LCO_CO	0.587	1.729	1.14

Table S2. Quantitative phase analysis of the LCO_CO composite determined by Rietveld refinement (WPPF).

Sample	LCO [wt.%]	CO [wt.%]
LCO_CO	67.3	32.7

Table S3. Elemental compositions (at.%) of CO, LCO, and LCO_CO composite catalysts determined by energy-dispersive X-ray spectroscopy (EDS).

**Sample**	**Co [at.%]**	**O [at.%]**	**La [at.%]**
**CO**	31.0	69.0	-
**LCO**	15.1	68.57	16.3
**LCO_CO**	25.0	70.3	4.8

Table S4. Double-layer capacitance (C_{dl}) values of CO, LCO, and LCO_CO composite catalysts derived from cyclic voltammetry measurements at various scan rates.

	(a) CO	(b) LCO	(c) LCO_CO
C_{dl} (mF)	0.3312	0.3076	0.2964

Table S5. Comparison of bifunctional catalytic activities (ORR and OER) of the LCO_CO composite with recently reported catalysts.

Catalyst	Electrolyte	$E_{1/2}$ (V)	η_{10} (V)	ΔE (V)	Year	Ref.
**LCO_CO**	0.1 M KOH	0.587	1.729	1.14	2026	This work
**$La(5B_{0.2})O_3$**	1 M KOH	0.723	1.681	0.96	2025	1
**LSCO**	0.1 M / 1 M KOH	0.85	1.69	0.96	2025	2
**LSF0.95/C**	0.1 M KOH	0.66	1.70	1.04	2021	3
**$CoFe_2O_4$/NCNTs**	0.1 M KOH	0.80	1.65	0.85	2019	4
**$(FeCrCuNiMn)_3O_4$/3D-G**	1 M KOH	0.837	1.589	0.75	2025	5

References:

1 G. Fu, R. Hou, L. Sun, S. Liu, H. Sun, Z. Shao and W. Jung, Rational regulation of high-entropy perovskite oxides through hole doping for efficient oxygen electrocatalysis, *ACS Appl. Mater. Interfaces*, 2025, **17**(5), 7860–7869. DOI: 10.1021/acsami.4c20338.

2 Y. Ali, S. Dwivedi, M. S. Alivand, A. Sanjid, A. Tanksale and P. Chakraborty Banerjee, Highly stable and porous triple perovskite oxide as a bifunctional electrocatalyst for rechargeable Zn-air batteries, *Chem. Eng. J.*, 2025, **516**, 163928. DOI: 10.1016/j.cej.2025.163928.

3 L. Luo, Z. Liu and Z. Wang, $(La_{0.65}Sr_{0.3})_{0.95}FeO_3$-δ perovskite with high oxygen vacancy as efficient bifunctional electrocatalysts for Zn–air batteries, *RSC Adv.*, 2021, **11**(62), 38977–38981. DOI: 10.1039/D1RA07920D.

4 X.-T. Wang, T. Ouyang, L. Wang, J.-H. Zhong, T. Ma and Z.-Q. Liu, Surface-interface engineering of hierarchical $CoFe_2O_4$/Ni-Fe layered double hydroxide nanowires for efficient oxygen evolution, *Angew. Chem., Int. Ed.*, 2019, **58**(38), 13291–13296. DOI: 10.1002/anie.201905448.

5 W. Li, Y. Wang, X. Sun, Y. Liang, J. Wang, L. Zhang and H. Zhang, High-entropy spinel oxides with oxygen-rich vacancies improve the catalytic performance for low-temperature flexible zinc-air batteries, *ACS Sustainable Chem. Eng.*, 2025, **13**(39), 16459–16473. DOI: 10.1021/acssuschemeng.5c05910.
